# Expression of RAC 3, a steroid hormone receptor co-activator in prostate cancer

**DOI:** 10.1054/bjoc.2001.2179

**Published:** 2001-12

**Authors:** V J Gnanapragasam, H Y Leung, A S Pulimood, D E Neal, C N Robson

**Affiliations:** Prostate Research Group, School of Surgical Sciences, University of Newcastle upon Tyne, Framlington Place, Newcastle upon Tyne, NE2 4HH, UK

**Keywords:** RAC 3, co-activator, androgen receptor, prostate cancer, survival outcome

## Abstract

RAC 3, one of the p160 family of co-activators is known to enhance the transcriptional activity of a number of steroid receptors. As co-activators are also known to enhance androgen receptor (AR) activity, we investigated the role of RAC 3 in the context of prostate cancer. In prostate cancer cell lines, we found variable levels of the RAC 3 protein with highest expression seen in AR-positive LNCaP cells, moderate expression in AR-negative PC 3 cells and low-level expression in AR-negative DU 145 cells. Immuno-precipitation studies showed that endogenous RAC 3 interacted with the AR in vivo and transfection assays confirmed that RAC 3 enhanced AR transcriptional activity. In clinical prostate tissue, we found strong RAC 3 mRNA expression and immuno-histochemistry demonstrated that in benign tissue, the protein was expressed predominantly in luminal cells, while in primary malignant epithelium it was more homogeneously expressed. In a series of 37 patients, the levels of RAC 3 expression correlated significantly with tumour grade (*P* = 0.01) and stage of disease (*P* = 0.03) but not with serum PSA levels. In addition moderate or high RAC 3 expression was associated with poorer disease-specific survival (*P* = 0.03). We conclude that RAC 3 is an important co-activator of the AR in the prostate and may have an important role in the progression of prostate cancer. © 2001 Cancer Research Campaign http://www.bjcancer.com


*British Journal of Cancer* (2001) **85**(12), 1928–1936
© 2001 Cancer Research Campaign
doi: 10.1054/ bjoc.2001.2179, available online at http://www.idealibrary.com on IDEAL

### Expression of RAC 3, a steroid hormone receptor co-activator in prostate cancer

**VJ Gnanapragasam, HY Leung, AS Pulimood, DE Neal and CN Robson**

Prostate Research Group, School of Surgical Sciences, University of Newcastle upon Tyne, Framlington Place, Newcastle upon Tyne NE2 4HH, UK

**Summary** RAC 3, one of the p160 family of co-activators is known to enhance the transcriptional activity of a number of steroid receptors. As co-activators are also known to enhance androgen receptor (AR) activity, we investigated the role of RAC 3 in the context of prostate cancer. In prostate cancer cell lines, we found variable levels of the RAC 3 protein with highest expression seen in AR-positive LNCaP cells, moderate expression in AR-negative PC 3 cells and low-level expression in AR-negative DU 145 cells. Immuno-precipitation studies showed that endogenous RAC 3 interacted with the AR in vivo and transfection assays confirmed that RAC 3 enhanced AR transcriptional activity. In clinical prostate tissue, we found strong RAC 3 mRNA expression and immuno-histochemistry demonstrated that in benign tissue, the protein was expressed predominantly in luminal cells, while in primary malignant epithelium it was more homogeneously expressed. In a series of 37 patients, the levels of RAC 3 expression correlated significantly with tumour grade ($P = 0.01$) and stage of disease ($P = 0.03$) but not with serum PSA levels. In addition moderate or high RAC 3 expression was associated with poorer disease-specific survival ($P = 0.03$). We conclude that RAC 3 is an important co-activator of the AR in the prostate and may have an important role in the progression of prostate cancer. © 2001 Cancer Research Campaign http://www.bjcancer.com

**Keywords**: RAC 3; co-activator; androgen receptor; prostate cancer; survival outcome

Prostate cancer growth is regulated in part by androgens and hormonal ablation in the form of surgical or chemical castration is the mainstay of treatment for men with advanced disease. However, such treatment is palliative and eventually most tumours become androgen-independent. New treatments are required to treat androgen-independent disease. One method to identify new therapeutic targets is to study molecular pathways involved in androgen receptor (AR)-mediated signalling.

Targeting and subsequent recruitment to the basal transcription machinery by steroid hormone receptors (SHR) is facilitated by sets of co-regulating proteins. Since the identification of the homologous SW1/SNF family of proteins in yeast, co-factors of SHRs have increased in number and complexity (Horwitz et al, 1996). These co-regulators; either co-activators or co-repressors, appear to be crucial for the assembly of the pre-initiation complex and recruitment of RNA polymerase II to the translation start site. The ‘squelching’ or competitive effect that different SHR families are able to exert on each other is thought to be due in part to competition for these factors (Meyer et al, 1989, 1992). Promiscuity of co-activators between different SHR systems mean that no co-activator has been specifically associated with a particular SHR. In vitro work has shown that certain co-activators can enhance AR activity 10–30 fold (Yeh and Chang 1996; Aarnisalo et al, 1998; Kang et al, 1999).

RAC 3, encoded on chromosome 20q12, is a 155 kDa protein that is a member of the p160 family of steroid receptor co-activators which include TIF 2 and SRC 1 (Li et al, 1997). It has also been designated as AIB 1 (amplified in breast 1), ACTR, TRAM 1, p/CIP, SRC 3 and in the Human Gene Nomenclature database as NCOA 3. For this manuscript we will hereafter refer to the gene as RAC 3. It was first identified in a yeast 2 hybrid system using the retinoic acid receptor as bait screening a brain cDNA library (Li et al, 1997). In this study, RAC 3 was found to be a co-activator of the retinoic acid receptor and enhanced its transcriptional activity. In the same year, Anzick et al (1997) identified RAC 3 (designated as AIB 1) as an amplified gene in breast cancer. They found that in oestrogen receptor (ER)-positive breast cancer cell lines, RAC 3 was amplified by greater than 20 fold. Furthermore, 10/105 primary breast tumour biopsies also demonstrated similar gene amplification. Subsequent tissue in situ hybridisation showed that RAC 3 expression at the transcript level was increased in tumour as compared to normal mammary epithelium in 58% of cases studied. In *in vitro* studies, RAC 3 was shown to enhance ER-dependent transcription by up to 3-fold. Bautista et al (1998) also demonstrated that RAC 3 amplification correlated with both tumour size and ER and progesterone receptor (PR) positivity in primary breast cancers. It has further been shown that endogenous RAC 3 interacts with the human ER in a ligand-dependent manner in MCF-7 breast cancer cells (Tikkanen et al, 2000). Interestingly the RAC 3 gene has also been found to be amplified in pancreatic cancers and primary gastric cancers suggesting that RAC 3 is functional in non-primarily steroid-dependent organs (Ghadimi et al, 1999; Sakakura et al, 2000).

RAC 3 over-expression in breast cancer and its ability to increase ER-dependent transcription suggests a role in tumour progression that involves interaction with the ER. In the light of similarities between prostate and breast cancer biology, and the fact that RAC 3 has been recently identified as an AR interacting protein (Tan et al, 2000), we proposed to examined the role of RAC 3 in the prostate. Our goal was to determine if RAC 3 interacted

*Received 4 June 2001*
*Revised 10 September 2001*
*Accepted 20 September 2001*

*Correspondence to:* CN Robson functionally with the human AR and if RAC 3 was expressed in prostate cancer cell lines. We then investigated expression in clinical prostate material and the possible relationship between expression levels and disease progression.

### MATERIALS AND METHODS

### Antibodies

Goat polyclonal anti-RAC 3 (Santa Cruz, Biotech, USA) was selected because of specific binding to the amino terminus of the co-activator and was used in Western blots and immuno-precipitation (IP). Also for IP and for immuno-staining, a mouse monoclonal antibody (Affinity Bioreagents, Cambridge, UK) was selected. This antibody was constructed from the fusion of a myeloma cell line to splenocytes from mice immunised with a GST fusion protein encoding residues 605–1294 of human RAC 3 (mapping to the C terminus of the protein). Mouse anti-human AR antibody was obtained from Pharmingen (USA).

### Western analysis

The prostate cancer cell lines: LNCaP, DU 145 and PC 3, the breast cancer cell line MCF-7 and the monkey kidney cell line Cos 7, were cultured in RPMI 1640 media (Life Technologies, USA) supplemented with 10% fetal calf serum (FCS) in 90-mm Petri dishes (Corning, USA) to near confluence. Cells were washed with phosphate-buffered saline (PBS) and harvested with SDS-loading buffer (0.125M Tris pH 6.8, 2% sodium dodecylsulfate, 10% glycerol, 10% β mercaptoethanol and 0.01% bromophenol blue). Cell lysates were stored at minus 70˚C. Normal human kidney lysate was obtained by suspension of crushed whole kidney sample in SDS buffer. 30 µl aliquots (approximately 100 µg) were subjected to 10% SDS-PAGE before protein transfer to nitrocellulose membranes (Hybond C, Amersham, UK). Filters were blocked with 5% dried milk at room temperature for 1 hour. Goat polyclonal RAC 3 antibody (1 µg $ml^{-1}$) was incubated with filters in sealed bags at room temperature for 1 hour. Filters were then incubated with the appropriate secondary antibody (horseradish peroxidase conjugated) before visualisation with ECL reagents (Amersham, UK) and exposed to autoradiographic film. Filters were stripped with 2% SDS buffer (100 mM β mercaptoethanol, 62.5 mM Tris HCl pH 6.7) incubated at 50˚C for 30 minutes and then re-probed with 0.5 µg $ml^{-1}$ of mouse monoclonal AR antibody. Loading was checked by re-stripping the filter and probing for α tubulin (Sigma, USA).

### Immuno-precipitation

LNCaP cells cultured in media with 10% dextran-coated charcoal steroid-depleted serum (DCC-FCS), DCC-FCS supplemented with 10 nM mibolerone (a synthetic androgen) or in full media as described above were harvested with PBS containing 0.02% EDTA solution, spun at 400 ***g*** for 5 minutes and the supernatant discarded. Cell pellets were re-suspended twice in 1 ml of lysis buffer (50 mM Tris pH 7.5, 150 mM NaCl, 0.2 mM $Na_3VO_4$, 1 mM DTT and 25 µg $ml^{-1}$ each of Aprotinin, Leupeptin, Pepstatin and PMSF). 20 µl of pre-prepared protein G sepharose (PGS) was added and the mix incubated at 4˚C for 2–4 hours. Mouse monoclonal AR antibody (0.5 µg $ml^{-1}$) or goat polyclonal RAC 3 antibody (0.2 µg $ml^{-1}$) was added and samples incubated overnight at 4˚C. Following this, a further 20 µ1 of PGS was added to each sample and incubated for 1 hour at room temperature. The samples were spun down and the supernatant discarded. The resultant pellet was washed in PBS-Triton with increasing stringency of 1 × Wash 1 (PBS, 0.2% Triton–100, 350 mM NaCl) and 2 × Wash 2 (PBS, 0.2% Triton–100). Pellets were re-suspended in 40 µl of SDS sample buffer and resolved by SDS-PAGE. Western analysis was then performed using the appropriate antibody. Controls of cell extract only and antibody only were taken through the immuno-precipitation steps and probed simultaneously.

### Cell culture and transfection assays

The following mammalian expression plasmids were kindly received; pCMX.RAC 3 full length incorporating a FLAG tag (Dr Don Chen, Massachusetts University, USA) and pSG5.ELE 1α (Dr B Peeters, University of Leuven, Belgium). pPSA.1uc, a reporter construct containing 2 androgen response elements and human AR cloned into the pCDNA3 vector have been previously described (Brady et al, 1999). Empty pSG5 vector was used to normalise the amount of DNA transfected in each experiment. DU 145 cells were seeded at 25 000 cells $well^{-1}$ in a 24-well plate (Corning). These were then transfected using Superfect (Qiagen) for a period of 2 hours (50 ng pCDNA3.AR, 125 ng pPSA.1uc, 200 ng pCMV.βgal and 200 ng pCMX.RAC 3, pSG5.Ele1α (a known AR co-activator) or empty pSG5) and then washed with PBS. Cos 7 cells were seeded at 20 000 cells $well^{-1}$ in a 24-well plate. These were transfected using Superfect for a period of 2 hours (50 ng pCDNA3.AR, 125 ng pPSA.1uc, 200 ng pCMV.βgal and 200 ng pCMX.RAC 3 or empty pSG5) and then washed with PBS. DCC FCS media only or supplemented with 10 nM mibolerone was then added and the cells incubated for 36–40 hours. Cells were harvested and assayed for luciferase activity according to manufacturers recommendations (Promega, UK) for 15–60 minutes. Luciferase activity was corrected for transfection efficiency by the corresponding β-galactosidase activity. β-galactosidase assays were typically performed in a 96-well plate (Corning, USA) as follows: sample extracts were incubated with an equal volume of β-galactosidase 2 × assay buffer (Promega, UK) and the reaction terminated with the addition of 1.5 volumes of 1 M sodium carbonate. $A_{420}$ values were then obtained using an MR5000 plate reader (Dynatech, UK) and activity calculated.

### In situ hybridization

Plasmid pT7T3D containing cDNA encoding 957 bp of RAC 3 was obtained from the I.M.A.G.E consortium (Clone ID 1256276, Acc No AA738120). The plasmid was purified and sequenced prior to use and verified against the published sequence of RAC 3 (Acc No AF012108). This clone mapped to the 3′ end of the gene and showed 99% homology using the NCBI BLAST program. Plasmid DNA was linearised with the restriction enzymes Not I or Eco RI. In vitro transcription was carried out to generate the anti-sense and sense probes using RNA polymerase. Digoxigenin–11-2′ deoxyuridine 5′-triphosphate was incorporated into the riboprobes (Boehringer, UK). RNA dot blot was used to quantify the amount of probe obtained. 50 µl of hybridisation mix (50% formamide, 4 × SSC, 1 × Denharts, 125 µg $ml^{-1}$ of tRNA) at a concentration of 0.5 ng riboprobe $µl^{-1}$ was added to each section and a coverslip applied. Hybridisation was carried out in a sealed

© 2001 Cancer Research Campaign humidification chamber at 52˚C overnight. A 1 in 500 dilution of alkaline phosphatase conjugated sheep anti-digoxigenin serum (Boehringer, UK) was incubated with the sections for 2 hours to detect bound digoxigenin-labelled probe. Signals were visualised by incubation with 5 bromo-4 chloro-3 indolyl phosphate and nitroblue tetrazolium for 18 hours in darkness. Signals were described as present or absent. All section were counter-stained with haematoxylin which identified the cellular architecture.

### Immuno-histochemistry

Immuno-staining was performed using a standard biotin–avidin–peroxidase method with either the mouse monoclonal or goat polyclonal antibodies described above. Bound antibody was detected with biotinylated anti-mouse or anti-goat immuno-globulin and diaminobenzidine tetrahydrochloride was used to generate signals. Sections incubated without any primary antibody were employed as negative controls and failed to exhibit any staining. Liver and kidney section were also used as negative controls.

### Patients and tissue

Men with newly diagnosed prostate cancer confirmed histologically were identified from a pathology department database. All patients had initially presented with bladder outflow obstruction and required transurethral resection of the prostate. 37 men (mean age 74, range 56–82 years) who had not had prior hormone manipulation and had continuous follow up till death or a minimum of 10 years were selected and studied for RAC 3 expression. In this cohort of patients, 27 went on to have hormonal manipulation (15 bilateral subcapsular orchidectomy, 12 anti-androgen therapy) and 1 patient went on to have a radical prostatectomy. Patients stage (TNM classification), tumour grade (Gleason sum score), PSA at diagnosis and survival was recorded. All patients were followed up in a standard manner with 3–6 monthly clinic appointments. 6 cases of benign prostatic hyperplasia (BPH) were also examined. Section of liver and kidney were obtained from archival material. Following formalin fixation and embedding in paraffin, 5 µm sections were taken and mounted on glass slides.

### Statistical analysis

All sections were studied independently by 2 observers without prior knowledge of the clinical details. 5 sections were previewed and inter-observer agreement obtained prior to evaluation of the whole series. Intensity of staining of RAC 3 in prostate cancer epithelium was graded as weak (+), moderate (++) or strong (+++). There were no non-staining cases in this series. Where 2 or more signal intensities were present in one slide, the intensity with greater than 50% area of staining was taken as the score for that section. Where there was discrepancy in the scoring, both observers reviewed the section together and a consensus reached. Differences in expression of RAC 3 protein in relation to clinical parameters were examined using the $\chi^2$ and Fishers exact test. Survival outcome in 36 patients (excluding the 1 who had radical surgery) was analysed with a Kaplan–Meier plot and the log-rank test. Survival was defined as the months patients lived following diagnosis and only deaths directly ascribed to prostate cancer as recorded in the death registry were counted as terminal events. A $P$ value of < 0.05 was taken to indicate statistical significance.

### RESULTS

### RAC 3 is expressed in prostate cancer cell lines

Western analysis was performed on a panel of cancer cell lines to investigate protein expression. MCF-7 cells are known to express high levels of the protein while kidney has been demonstrated to not express RAC 3 at the transcript level. These were used as positive and negative controls respectively. RAC 3 polyclonal goat antibody demonstrated strong signals in the MCF-7 cell line while AR-positive LNCaP cells expressed the highest levels of RAC 3 among the prostate cancer cell lines. AR-negative PC 3 cells also expressed the protein while in similarly AR-negative DU 145 cells, RAC 3 protein was barely detectable. No signal was apparent in either normal human kidney lysate or monkey kidney Cos 7 cells (Figure 1). AR expression was examined on the same filters by stripping and re-probing with a mouse monoclonal AR antibody. This demonstrated AR expression in LNCaP cells and lower levels in MCF-7 cells. Loading was verified by probing with an α-tubulin-specific antibody. These results demonstrate that RAC 3 is expressed in prostate cancer cell lines and remains expressed in AR-independent cell lines though the levels vary depending on the cell line. AR-positive prostate cancer cells however do appear to express the highest amounts of RAC 3.

### RAC 3 interacts with the AR and enhances transcriptional activity

To investigate in vivo interaction, immuno-precipitation was performed utilising cultured LNCaP cells in the presence or absence of androgens. Androgen receptor was immuno-precipitated from cell lysates, resolved on SDS PAGE and probed with RAC 3 antibody and the converse experiment performed. In these studies RAC 3 interacted with the AR (Figure 2A and 2B). This interaction was strongest in the presence of androgens (mibolerone 10 nM–lane 1) and weakest when the ligand was absent (lane 3). This suggests that the strength of AR-RAC 3 interaction is ligand-dependent. No band was detected in lanes with cell lysate only (lane 4) or with antibody only (lane 5). To confirm the functional importance of this interaction we performed in vitro transcription studies. In DU145 cells co-transfected with AR and the PSA promoter, RAC 3 enhanced PSA promoter activity by up to 3-fold above levels induced by androgens only and by up to 5-fold above

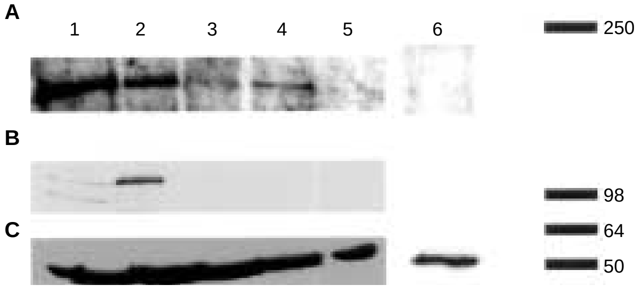

**Figure 1** RAC 3 is expressed in prostate cancer cell lines. MCF-7 (1) cells (positive control), LNCaP (2), DU 145 (3), PC 3 (4), Cos 7 (5) cells were run out concurrently. (**A**) Western analysis with goat polyclonal anti-human RAC 3, whole human kidney lysate (6) (negative control) did not express RAC 3. (**B**) Same filter stripped and re-probed with mouse monoclonal anti-human AR. (**C**) Same filter as well as whole human kidney lysate blot stripped and re-probed with anti-α-tubulin antibody

 *© 2001 Cancer Research Campaign*

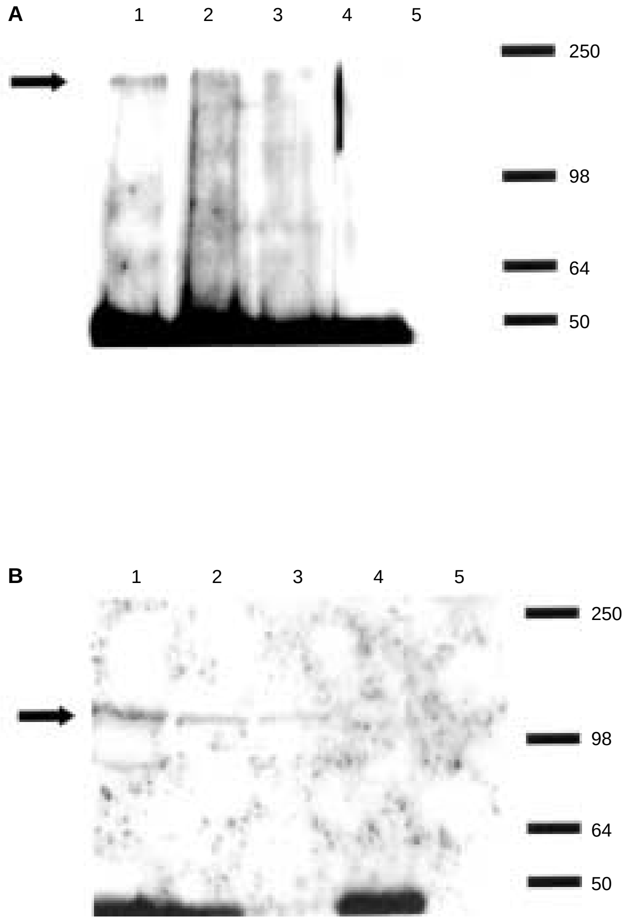

**Figure 2** RAC 3 interacts with the AR in vivo in a ligand-dependent manner. (**A**) Immuno-precipitation performed on LNCaP cell lysate using goat polyclonal anti-human RAC 3 and then probed with mouse monoclonal anti-human AR. (**B**) Immuno-precipitation performed on LNCaP cell lysate using mouse monoclonal anti-human AR and then probed with goat polyclonal anti RAC 3. Lane 1 – cells cultured in DCC with 10 nM mibolerone. Lane 2 – cells cultured in full media. Lane 3 – cells cultured in DCC only. Lane 4–cell lysate only with no IP antibody. Lane 5 – IP antibody only with no cell lysate. The immuno-precipitation band is indicated with an arrow in each case

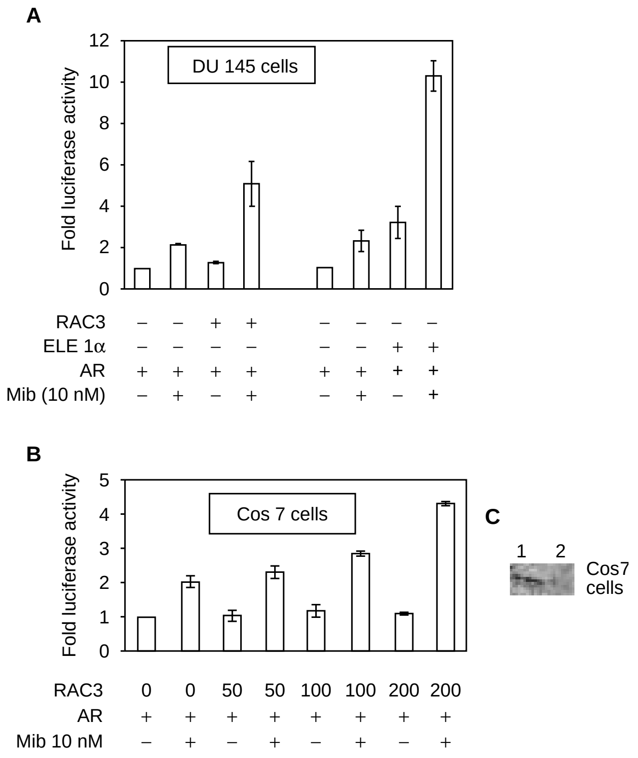

**Figure 3** RAC 3 is a co-activator of the AR in prostate cells. Luciferase results are expressed as fold induction of luciferase activity from basal levels corrected for transfection efficiency. (**A**) DU 145 cells were transfected with 50 ng of pcDNA3.AR, 125 ng pPSA.luc, 200 ng pCMV.βgal and 200 ng pCMX.RAC 3, pSG5.Ele 1α (a known AR co-activator) or empty pSG5 vector per well of a 24 well plate. Cells were then cultured in the presence or absence of the synthetic androgen mibolerone. Results shown are the mean and SD of 3 experiments, each performed in triplicate. (**B**) Cos 7 cells were transfected with 50 ng pcDNA3. AR, 125 ng pPSA.luc, 200 ng pCMV.βgal and 50–200 ng pCMX.RAC 3 per well of a 24 well plate. Results shown are the mean and SD of 2 experiments, each performed in triplicate.
(**C**) Presence of RAC 3 in transfected Cos 7 cells was confirmed by Western blot against the FLAG tag of the pCMX.RAC 3 vector. Lane 1 RAC 3 transfected Cos 7 cells (100 ng pCMX.RAC 3), Lane 2 untransfected cells basal PSA promoter activity (Figure 3A) in the presence of mibolerone. Similar studies with the ELE 1α co-activator demonstrated 5-fold enhancement of reporter activity above levels found following the transfection of AR and application of mibolerone and up to 10-fold above basal PSA promoter activity. Unlike RAC 3 however, ELE 1α appeared to have a mild inductive effect on AR transactivation even in the absence of mibolerone. In duplicate transfections in Cos 7 cells, similar induction of the AR was observed and in addition the effect of RAC 3 on AR transcription was seen to be dose-dependent (Figure 3B). To confirm that the RAC 3 plasmid was expressed following transfection, Western blot was performed for the FLAG tag on the pCMX.RAC 3 plasmid. This confirmed high level expression of RAC 3 as compared to un-transfected cells (Figure 3C).

### RAC 3 is expressed in clinical biopsies at the transcript level

Paraffin-embedded sections of benign prostate tissue was subjected to RNA in situ hybridisation. Anti-sense probes (conferring an intense blue stain) generated cytoplasmic signals in these (Figure 4A) while sense probes failed to generate a signal (Figure 4B) showing only the nuclear counter stain. The anti-sense probe hybridised strongly to epithelial tissue with little or no signals in the stroma. This confirmed expression of RAC 3 at the mRNA level in prostate tissue and suggests that it is expressed predominantly in epithelium. Sections of prostate cancer similarly probed for RAC 3 also demonstrated similar epithelial signals with little or no stromal expression (data not shown).

### RAC 3 protein is differentially expressed in BPH and malignant epithelium

The mouse monoclonal antibody used for immuno-histochemistry has not previously been validated for this application. Because of this, we checked the staining pattern seen with a goat polyclonal antibody raised against a different region of the protein. This antibody was further validated by confirming specificity in detecting RAC 3 on Western blots with the control cell line MCF-7. Immuno-histochemistry showed consistent patterns of expression in BPH and malignant epithelium. In BPH there was generally weak staining for RAC 3 (Table 1), in addition staining was seen predominantly in luminal cells rather than basal cells (Figure 4C). Staining for the AR in adjacent slides of BPH showed that these

*© 2001 Cancer Research Campaign* 

**Table 1** RAC 3 expression is associated with severity of disease. Clinical details of the patients studied with either BPH or prostate cancer and concomitant RAC 3 expression. Patients with prostate cancer were analysed by stage, Gleason sum score and PSA level at diagnosis. A *P* value of < 0.05 was taken as significant. Treatments received post TURP are also included

| Clinical details | RAC 3 expression | | | Total | *P* value |
|---|---|---|---|---|---|
| | + | ++ | +++ | | |
| BPH | 4 | 2 | 0 | 6 | — |
| Prostate cancer | | | | | |
| Stage[a] ($n$ = 37) | | | | | |
| T1–T2 | 7 | 10 | 6 | 23 | |
| T3–T4 | 1 | 5 | 8 | 14 | 0.03 |
| Grade[b] ($n$ = 37) | | | | | |
| 4–6 | 5 | 1 | 2 | 8 | |
| 7–10 | 3 | 13 | 13 | 29 | 0.01 |
| PSA ($n$ = 24) | 70.9 | 34.2 | 89.9 | | |
| Mean values at diagnosis in ng $ml^{-1}$ | (25.7–154) | (2.8–200) | (2.7–240) | — | NS |
| Post TURP treatment ($n$ = 37) | | | | | |
| None | 3 | 4 | 1 | 8 | |
| BSO[c] | 1 | 5 | 9 | 15 | |
| Anti-androgens | 2 | 6 | 4 | 14 | |
| Radical surgery | 1 | 0 | 0 | 0 | — |

[a]Stage of disease is defined as organ confined (T1 and T2) or spread through and beyond the prostate (T3 and T4) (TNM staging system). [b]Gleason grade is the sum score of the 2 predominant grades of cancer in a section (range 2 to 10) and indicates increasing severity of disease. [c]Bilateral subcapsular orchidectomy.

luminal cells were AR-positive while basal cells were AR-negative (Figure 4D). In cancer there was more homogeneous staining of epithelial cells and this pattern was apparent regardless of the grade of the disease with variations only in the intensity of staining (Figure 4E–H). In both benign and malignant epithelium, RAC 3 appeared to be distributed in the cytoplasmic and nuclear compartments. In many cases we observed that RAC 3 signals were most evident in the cytoplasm. Stromal signals were equivocally weak or non-existent in BPH and cancer corresponding to the signal pattern seen at the mRNA level. Sections of liver and kidney were also stained as putative-negative controls. As suggested by previous RNA studies, neither tissue expressed the RAC 3 protein under standard immuno-histochemical conditions (Figure 4I–J). Sections of prostate cancer with no primary antibody failed to exhibit any staining (Figure 4K).

### Expression of RAC 3 is associated with tumour grade, stage and outcome but not with PSA

A preliminary series of 37 prostate-cancer patients were studied to investigate the relationship between RAC 3 staining and disease progression. Pathological grading of prostate cancer is currently done using the Gleason sum score. This is the sum of the 2 most prominent grades of cancer (range 1–5) seen in a specimen of tissue. In this series Gleason sum score was associated with strong expression of RAC 3 when the 3 levels of staining intensity were looked at separately ($P$ = 0.01, $n$ = 37; $\chi^2$) (Table 1). This association was further strengthened when the moderate and strong signals were grouped together ($P$ = 0.0056, Fisher's exact test). When tumours were divided into T1/T2 (organ confined) or T3/T4 (non-confined) disease, tumour stage was found to be significantly associated with signal intensity ($P$ = 0.03, $n$ = 37, $\chi^2$) (Table 1). Survival outcome analysis in 36 cases (excluding the patient who had received radical surgery) suggested that moderate or strong expression was associated with a poorer outcome (Figure 5) when compared to weak expression (log-rank test $P$ = 0.03). There was, however, no difference in survival outcome when the moderate- and strong-expressing groups were compared separately ($P$ = 0.09) or when the weak and moderate groups were pooled together ($P$ = 0.19). These preliminary results suggest that the level of expression of this co-activator in malignant prostate epithelium has an association with the severity of clinical disease. PSA at diagnosis was found to bear no relationship to RAC 3 expression.

### DISCUSSION

In hormone refractory prostate cancer, the role of the AR is uncertain. The cell lines PC 3 and DU 145, for instance, are highly metastatic and do not express the AR (Culig et al, 1993). In contrast it is well known that following androgen ablation serum PSA, which is androgen regulated, begins to rise before clinical evidence of recurrent disease is detected. Using immuno-histochemistry, many studies have shown that the AR continues to be expressed in hormone-resistant and metastatic cancers (Van der Kwast et al, 1991; Sadi et al, 1991; Hobisch et al, 1996). In the prostate CWR22 xenograft model, AR mRNA and protein levels in relapse tumours increase as hormone independence occurs (Wainstein et al, 1994; Gregory et al, 1995). Gene amplification of the AR has also been reported in hormone-relapse prostate cancers (Cher et al, 1996) and Koivisto et al (1997) showed that AR mRNA levels are also increased in this group of patients. It is not possible at present however to manipulate this resurgent AR to control cancer progression.

The cytoplasmic AR has been shown to have a signalling cascade that is independent of nuclear translocation (Peterziel et al, 1999). It is possible that this pathway becomes increasingly

 *© 2001 Cancer Research Campaign*

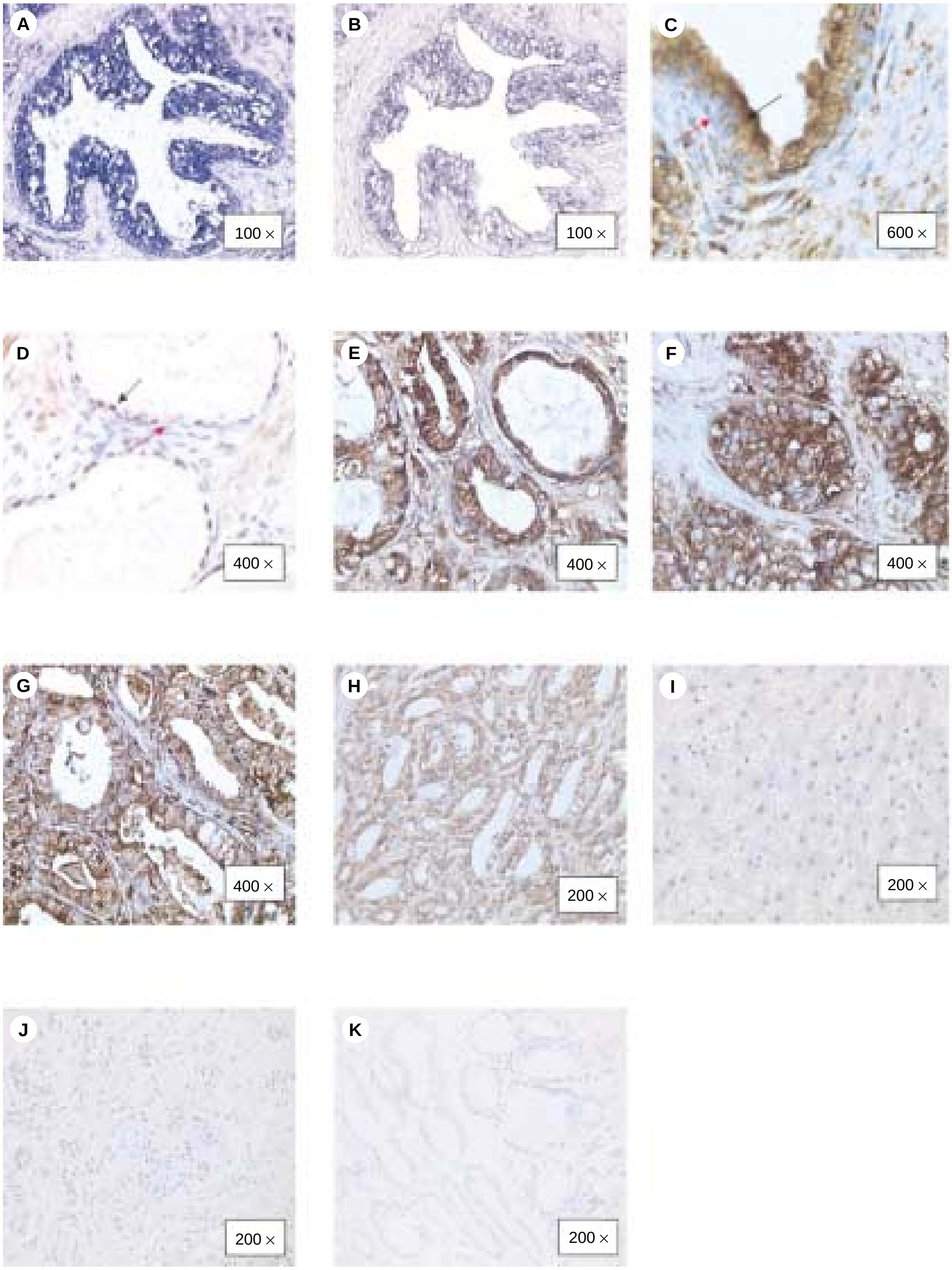

**Figure 4** RAC 3 expression in clinical prostate tissue. (**A**,**B**) Sections of prostate tissue hybridised with antisense and sense RAC 3 mRNA riboprobes respectively. (**C**) Section of BPH stained for RAC 3 protein, prominent luminal cell staining (black arrow) is seen with relative sparing of the basal cells (red arrow). (**D**) Section of BPH tissue stained for the androgen receptor showing expression in luminal cells only (black arrow) and negative staining in basal cells (red arrow). (**E**,**F**) High-grade prostate cancer cells with strong staining for RAC 3 protein. (**G**) Moderately differentiated cancer cells showing moderate staining for RAC 3 protein. (**H**) Prostate cancer cells showing weak staining for RAC 3. (**I**,**J**) Sections of human liver and kidney respectively with negative immuno-staining for RAC 3 protein. (**K**) Section of prostate cancer with no primary antibody showing negative signals effective when ligand-AR signalling is abrogated. Alternatively, constituent facilitators of AR nuclear signalling may be over-expressed. The co-activator CBP for instance has been shown to be a mediator of transcriptional interference between the AR and the AP–1/NFκB transcription factors suggesting that this co-activator may be an integrator between androgen-mediated and other signalling pathways (Aarnisalo et al, 1998; Wadgoanker et al, 1999). The role of co-activators in cancer remains elusive. Their

*© 2001 Cancer Research Campaign*

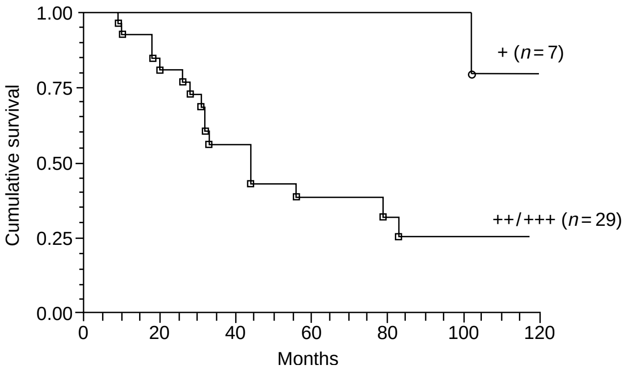

**Figure 5** RAC 3 expression is associated with disease-specific survival. RAC 3 staining was stratified as weak (+) or moderate (++) and strong (+++) for the purpose of survival analysis ($n$ = 36). Patients were followed up for a mean of 10 years or until death as a direct cause of prostate cancer. A Kaplan–Meier plot is shown constructed from data on disease specific survival (log-rank test $P$ = 0.03)

intracellular role and their *in vivo* interactions are increasing in complexity. As such, it is not possible to identify a particular co-activator as being responsible for a particular SHR's transcriptional activation. Promiscuity, even within the p160 co-activator family, was emphasized when SRC 1 knockout mice remained viable with few mature differences from normal mice (Xu et al, 1998). Nevertheless, some interesting studies have linked co-activators to cancer progression. CBP/p300 has been found to be mutated in cancer and may be involved in MDM 2 regulation of p53, a tumour suppressor (Scolnick et al, 1997; Wadgoanker et al, 1999). ARA 70, also designated ELE 1α, was initially identified as a putative AR-specific co-activator (Yeh and Chang 1996; Alen et al, 1999) but has been shown to be able to facilitate anti-androgen signalling to androgen response elements (ARE) thus postulating a mechanism for the so-called anti-androgen withdrawal syndrome (Miyamoto et al, 1998). Furthermore, 17-β estradiol has been shown to activate AR target genes via its interaction with the AR-ARA70 complex in the absence of androgens (Yeh et al, 1998). Levels of SRC 1, the first identified p160 co-activator, in a study of 21 breast cancers appeared to be able to predict case response to tamoxifen in patients with recurrent breast cancer (Berns et al, 1998). Certain members of the family of p160 co-activators possess histone acetyl transferase (HAT) activity and histone acetylation is thought to be an important prerequisite for transcriptional activity of many genes (Hassig and Schreiber 1997). It is possible that over-expression of HATs may therefore facilitate the activity of genes important in cancer progression. In acute myeloid leukaemia for example, the translocation t(8;16)(p11p13), fusing the MOZ protein to CBP, was found by cytogenetic analysis of the M4/M5 subtype of the disease. The authors suggest that the dominant MOZ-CBP fusion protein was mediating leukaemogenesis by abnormal levels of acetylation (Borrow et al, 1996).

RAC 3 possesses HAT activity and shares over 40% homology with SRC 1, it contains a well-conserved N terminus basic LHL and period-aryl hydrocarbon receptor-single minded (PAS) A and B domains (Li et al, 1997). Like SRC 1, RAC 3 contains a number of LXXLL motifs that interact with liganded receptors. RAC 3 unlike SRC 1 however, appears to be more important in embryonic development with genetic disruption of RAC 3 resulting in mice with dwarfism, delayed puberty, reduced female reproductive function and blunted mammary development (Xu et al, 2000). RAC 3's role as an ER co-activator has already been discussed but in addition to SHRs, RAC 3 has been shown to be a co-activator for the NFκB transcription factor (Werbajh et al, 2000). In this study, RAC 3 was competed for by both NFκB and the glucocorticoid receptor (GR) thus RAC 3 binding to NFκB was able to abrogate NFκB–GR transrepression. RAC 3 may also be involved in cell signalling. Font de Mora and Brown (2000) have recently shown that RAC 3 is phosphorylated by MAPK and MAPK signalling to the ER is known to stimulate ligand-independent receptor activity. In this context phosphorylated RAC 3 stimulated the recruitment of p300 and its associated histone acetyl transferase activity to the ER complex. The authors postulate that modulation of ER function by growth factors may therefore be mediated by MAPK phosphorylation of RAC 3. Other workers have also shown that RAC 3 interacts with the ubiquitous co-activator CBP (Li and Chen 1998) which raises the possibility of RAC 3 being an integrator or recruiter of other co-activators. RAC 3 expression has been identified at the RNA level in human heart, placenta, muscle, pancreas and prostate but not in lung, liver, brain or kidney suggesting some tissue specificity (Li and Chen, 1998; Fujimoto et al, 2001). Protein expression has also been confirmed in breast and ovarian cancer cell lines (Azorsa and Meltzer, 1999). The relative specificity of RAC 3 expression in human tissues and its apparent important role in breast cancer led us to believe that this co-activator may have a role in the prostate.

Using RT-PCR (reverse transcriptase-polymerase chain reaction), Fujimoto et al (2001) examined levels of RAC 3 in a panel of prostate cancer cell lines. They observed low levels of expression in DU 145 cells, moderate expression in LNCaP cells and high levels of expression in PC 3 cells. Conversely, a similar study by Nessler-Menardi et al (2000) again using RT-PCR, found very low levels of expression in LNCaP cells and moderate expression in DU 145 cells. These discrepancies may have arisen because RT-PCR is not inherently a quantitative technique. By Western analysis we observed most prominent expression of the RAC 3 protein in AR-positive LNCaP cells. Longer exposure of the blot confirmed weak expression in AR-negative DU 145 cells compared to moderate expression in AR-negative PC 3 cells and high expression in LNCaP cells. No signals were apparent in normal kidney lysate and the monkey kidney cell line Cos 7. We believe that this analysis of RAC 3 protein levels reflect the true functional abundance of the co-activator in these cell lines. Both PC 3 and DU 145 cells do not express AR and the presence and role of RAC 3, especially in PC3 cells, is intriguing.

LNCaP cells were predictably strongly positive for the AR while in MCF-7 cells we observed lower levels of AR expression and a smaller AR fragment (approximately 80 kDa) which probably represents a known truncated form of the receptor previously reported by other researchers (Wilson and Mcfhaul, 1994). In LNCap cells, immuno-precipitation experiments confirmed that endogenous AR and RAC 3 interacted physically at the protein level. This result is in keeping with previous findings by Tan et al (2000). In their study affinity matrix assays demonstrated that RAC 3 was able to bind to the AR-ligand-binding domain and joint AR N terminal and DNA-binding domains but not to the DNA-binding domain on its own. Our studies have also found that the strength of the AR-RAC 3 interaction is dependent on the presence of androgens.

Transcription studies demonstrated that this interaction was functional in an in vitro cell model (DU 145 and Cos 7 cells). The degree of transcriptional enhancement of the wild-type AR was not as great as that seen with ELE 1α nor that of other reported co-activators (Aarnisalo et al, 1998; Brady et al, 1999). Nevertheless,

 *© 2001 Cancer Research Campaign*

the degree of enhancement is in agreement with previous reports of RAC 3 co-activation (Li and Chen, 1998; Tan et al, 2000) and is similar to that reported for the ER (Anzick et al, 1997). Experiments in the LNCaP cell line with a native AR also showed moderate transcriptional enhancement by RAC 3 suggesting that the effect is not cell type-specific (data not shown). We also observed that ELE 1α in addition to enhancing liganded AR activity was able to enhance ligand-independent AR transactivation which did not occur in the presence of RAC 3. This result supports the earlier observation that AR-RAC 3 interaction is ligand-dependent. The AR–ELE 1α complex may also be responding to other ligands, as has been previously observed (Miyamoto et al, 1998; Yeh et al, 1998) which do not affect AR-RAC 3 interaction. While RAC 3 interacts with the AR and is able to enhance its transcriptional activity, the fact that it is expressed in AR-independent prostate cells and is over-expressed in non-SHR-dependent tissue suggests a supplementary role for RAC 3 in cancer progression that appears to be independent of the AR. Another mode of RAC 3 activity in prostate cancer cells may be via its well characterised interaction with the ER (Anzick et al, 1997; Tikkanen et al, 2000; Xu et al, 2000). The oestrogen receptor (ER) has been shown to be present in human prostate cancer and pre-malignant lesions (Bonkhoff et al, 1999), while the prostate cell lines LNCaP, DU 145 and PC 3 have been shown to express the β isoform of the receptor (Lau et al, 2000). From these studies, ER status appears to be an important factor in prostate cancer progression. It is possible that in addition to its co-activation of the AR, RAC3 may be involved in enhancing ER activity in prostate cancer cells.

In situ hybridisation confirmed strong expression of RAC 3 mRNA in epithelial cells with only few scattered signals in the stroma. This was confirmed by the immuno-histochemistry data which detected only a few scattered signals in the stroma. In clinical prostate tissue we found that protein expression of RAC 3 differed between benign and malignant prostate epithelium. In benign tissue more prominent staining was seen in luminal cells as compared to basal cells while in cancer, the staining was more homogeneous. This may be due to the fact that undifferentiated basal cells do not express the androgen receptor while luminal cells do. This selectivity is lost in malignant epithelium which is known to be of a luminal phenotype. Interestingly we found that RAC 3 was expressed in both the cytoplasm and nucleus of cells. While this may represent an artifact of the fixative procedure, it could also be due to RAC 3 interacting with the AR in both compartments. Co-localisation studies (AR and RAC 3) would help to clarify this. Interestingly, studies involving other AR co-activators in the prostate e.g. FHL 2 have also demonstrated both cytoplasmic and nuclear staining (Muller et al, 2000).

To correlate staining with the severity of disease we used a system of graded intensities. All our patients had 10 years or more follow up and Gleason sum scoring was performed by an experienced pathologist. Grade and stage of disease but not PSA at diagnosis, was found to correlate significantly with the intensity of RAC 3 staining. To investigate if expression RAC 3 had an association with clinical outcome, we proceeded to analyse signal intensities with respect to disease-specific survival. In this retrospective cohort, tumours expressing moderate or strong levels of RAC 3 appeared to have a poorer outcome. This association was lost however if the 3 signal intensities were examined separately. These initial results need to be verified with a larger study but is nevertheless suggestive of a role for over-expression of RAC 3 in prostate cancer progression.

In conclusion, we have shown evidence for an important role for RAC 3 in the prostate. We have demonstrated that this co-activator interacts with endogenous AR in a ligand-dependent manner and functionally enhances transcriptional activity in prostate cancer cells. We demonstrate expression of the protein in both androgen-dependent and -independent prostate cell lines and show that RAC 3 is expressed at the mRNA and protein level in prostate biopsies. Furthermore we found preliminary evidence that the expression differed between benign and malignant epithelium, and that moderate and strong staining for RAC 3 was associated with high-grade, late-stage disease and a poor outcome. Taken together these data suggest that RAC 3 is an important co-activator in prostate cancer and warrants further study to define its role in prostate cancer progression.

### ACKNOWLEDGEMENTS

This project was funded by the Cancer Research Campaign of the United Kingdom, grant no SP2503/0101. The first author holds a Research Fellowship for a Clinician granted by the Cancer Research Campaign.

### REFERENCES

Aarnisalo P, Palvimo JJ and Janne OA (1998) CREB-binding protein in androgen receptor mediated signaling. *Proc Natl Acad Sci USA* **95**: 2122–2127

Alen P, Claessens F, Schoenmakers E, Swinen JV, Verhoeven G, Rombauts W and Peeters B (1999) Interaction of the putative androgen receptor specific co-activator ARA70/ELE 1 α with multiple steroid receptors and identification of an internally deleted ELE 1 β isoform. *Mol Endo* **13**: 117–128

Anzick SL, Kononen J, Walker RL, Azorsa DO, Tanner MM, Guan X-Y, Sauter G, Kallioniemi O-P, Trent JM and Meltzer PS (1997) AIB 1, a steroid receptor co-activator amplified in breast and ovarian cancer. *Science* **277**: 965–968

Azorsa DO and Meltzer PS (1999) Production and characterization of monoclonal antibodies to the steroid receptor co-activator AIB 1. *Hybridoma* **18**: 281–287

Bautista S, Valles H, Walker RL, Anzick SL, Zeillinger R, Meltzer PS and Theillet C (1998) In breast cancer, amplification of the steroid receptor coactivator gene AIB1 is correlated with estrogen and progesterone receptor positivity. *Clin Cancer Res* **4**: 2925–2929

Berns EMJJ, van Staveren IL, Klijn JGM and Foekens JA (1998) Predictive value of SRC–1 for tamoxifen response of recurrent breast cancer. *Breast Cancer Res and Treat* **48**: 87–92

Bonkhoff H, Fixemer T, Hunsicker I and Remberger K (1999) Estrogen receptor expression in prostate cancer and premalignant prostatic lesions. *Am J Pathol* **155**: 641–647

Borrow J, Stanton VP, Andresen JM, Becher R, Behm FG, Chaganti RSK, Civin CI, Disteche C, Dube I, Frischauf AM, Horsman D, Mitelman F, Volinia S, Watmore AE and Housman DE (1996) The translocation t(8;16)(p11:p13) of acute myeloid leukemia fuses a putative acetyltransferase to the CREB binding protein. *Nature Gen* **14**: 33–41

Brady ME, Ozanne DM, Gaughan L, Waite I, Cook S, Neal DE and Robson CN (1999) Tip60 is a nuclear hormone receptor co-activator. *J Biol Chem* **274**: 17599–17560

Cher ML, Bova GS, Moore DH, Small EJ, Carroll PR, Pin SS, Epstein JI, Isaacs WB and Jensen RH (1996) Genetic alterations in untreated metastases and androgen-independent prostate cancer detected by comparative genomic hybridization and allelo-typing. *Cancer Res* **56**: 3091–3102

Culig Z, Klocker H, Eberle J, Kaspar F, Hobisch A, Cronauer MV and Bartsch G (1993) DNA sequence of the androgen receptor in prostatic tumour cell lines and tissue specimens assessed by means of the polymerase chain reaction. *Prostate* **22**: 11–22

Font de Mora J and Brown M (2000) AIB 1 is a conduit for kinase-mediated growth factor signaling to the estrogen receptor. *Mol Cell Biol* **20 (14)**: 5041–5047

Fujimoto N, Mizokami A, Harada S and Matsumoto T (2001) Different expression of androgen receptor co-activators in human prostate. *Urology* **58**: 289–294

Ghadimi BM, Schrock E, Walker RL, Wangsa D, Jauho A, Meltzer PS and Reid T (1999) Specific chromosomal aberrations and amplification of the AIB1 nuclear receptor co-activator gene in pancreatic carcinomas. *Am J Path* **154**: 525–536

© 2001 Cancer Research Campaign

Gregory CW, Sharief Y, Hamil KG, Hall SH, Pretlow TG, Mohler JL and French FS (1995) Apoptosis in an androgen-dependent xenograft model derived from a primary human prostatic carcinoma. *Mol Biol Cell* **6**: 1438 (Abstract)

Hassig CA and Schreiber SL (1997) Nuclear histone acetylases and deacetylases and transcriptional regulation: HATs off to HDACs. *Curr Opin Chem Biol* **1**: 300–308

Hobisch A, Culig Z, Radmayr C, Bartsch G, Klocker H and Hittmair A (1996) Androgen receptor status of lymph node metastases from prostate cancer. *Prostate* **28**: 129–135

Horwitz KB, Jackson TA, Bain DL, Richer JK, Takimoto GS and Tung LS (1996) Nuclear receptor co-activators and co-repressors. *Mol Endo* **10**: 1167–1177

Kang HY, Yeh S, Fujimoto N and Chang C (1999) Cloning and characterization of human prostate co-activator ARA 54, a novel protein that associates with the androgen receptor. *J Biol Chem* **274**: 8570–8576

Koivisto P, Kononen J, Palmberg C, Tammela T, Hyytinen E, Isola J, Trapman J, Cleujens K, Noordzij A, Visakorpi T and Kallioniemi OP (1997) Androgen receptor gene amplification: A possible molecular mechanism for androgen deprivation therapy failure in prostate cancer. *Cancer Res* **57**: 314–319

Lau KM, La Spina M, Long J and Ho SM (2000) Expression of estrogen receptor (ER)-alpha and ER-beta in normal and malignant prostatic epithelial cells: regulation by methylation and involvement in growth regulation. *Cancer Res* **60**: 3175–3182

Li H and Chen JD (1998) The Receptor Associated Co-activator 3 activates transcription through CREB binding protein recruitment and autoregulation. *J Biol Chem* **273**: 5948–5954

Li H, Gomes P and Chen JD (1997) RAC 3, a steroid/nuclear receptor-associated co-activator that is related to SRC 1 and TIF 2. *Proc Natl Acad Sci USA* **94**: 8479–8484

Meyer ME, Gronemeyer H, Turcotte B, Bocquel M-T, Tassest D and Chambon P (1989) Steroid hormone receptors compete for factors that mediate their enhancer function. *Cell* **57**: 433–442

Meyer ME, Quirin-Sricker C, Lerouge T, Bocquel M-T and Gronemeyer H (1992) A limiting factor mediates the differential activation of promoters by the human progesterone receptor isoforms. *J Biol Chem* **267**: 10882–10887

Miyamato H, Yeh S, Wilding G and Chang C (1998) Promotion of agonist activity of antiandrogens by the androgen receptor co-activator, ARA70, in human prostate cancer DU145 cells. *Proc Natl Acad Sci USA* **95**: 7379–7384

Muller JM, Isele U, Metzger E, Rempel A, Moser M, Pscherer A, Breyer T, Holubarsch C, Buettner R and Schule R (2000) FHL2, a novel tissue specific coactivator of the androgen receptor. *EMBO J* **19**: 359–369

Nessler-Menardi C, Jotova I, Culig Z, Eder I, Putz T, Bartsch G and Klocker H (2000) Expression of androgen receptor coregulatory proteins in prostate cancer and stromal cell culture models. *Prostate* **45**: 124–131

Peterziel H, Mink S, Schonert A, Becker M, Klocker H and Cato ACB (1999) Rapid signaling by androgen receptor in prostate cancer cells. *Oncogene* **18**: 6322–6329

Sadi MV, Walsh PC and Barracks ER (1991) Immuno-histochemical study of androgen receptors in metastatic prostate cancer-comparison of receptor content and response to hormonal therapy. *Cancer* **67**: 3057–3064

Sakakura C, Hagiwara A, Yasuoka R, Fujita Y, Nakanishi M, Masuda K, Kimura A, Makamura Y, Inazawa J, Abe T and Yamagishi H (2000) Amplification and over-expression of the AIB1 nuclear receptor in primary gastric cancers. *Int J Cancer* **89**: 217–223

Scolnick DM, Chehab NH, Stavridi ES, Lien MC, Caruso L, Moran E, Berger SL and Halazonetis TD (1997) CREB-binding protein and p300/CBP-associated factor are transcriptional co-activators of the p53 tumour suppressor protein. *Cancer Res* **57**: 3693–3696

Tan JA, Hall SH, Petrusz P and French FS (2000) Thyroid receptor activator molecule, TRAM–1, is an androgen receptor co-activator. *Endocrinology* **141(9)**: 3440–3450

Tikkanen MK, Carter DJ, Harris AM, Le HM, Azorsa DO, Meltzer PS and Murdoch FE (2000) Endogenously expressed estrogen receptor and coactivator AIB1 interact in MCF-7 human breast cancer cells. *Proc Natl Acad Sci USA* **97**: 12536–12540

Van der Kwast TH, Schalken J, Ruizveld de Winter JA, van Vroohoven CCJ, Mulder E, Boersma W and Trapman J (1991) Androgen receptors in endocrine-therapy-resistant human prostate cancer. *Int J Cancer* **48**: 189–193

Wadgoanker R and Collins T (1999) Murine Double minute (MDM 2) blocks p53 co-activator interaction, a new mechanism for inhibition of p53 dependent gene expression. *J Biol Chem* **274**: 13760–13767

Wadgoanker R, Phelps KM, Haque Z, Williams AJ, Silverman ES and Collins T (1999) CREB binding protein is a nuclear integrator of nuclear factor-κB and p53 signaling. *J Biol Chem* **274**: 1879–1886

Wainstein MA, He F, Robinson D, Kung H-J, Scwartz S, Giaconia JM, Edgehouse NL, Pretlow TP, Bodner DR, Kursh ED, Resnick MI, Seftel A and Pretlow TG (1994) CWR22:Androgen-dependent xenograft model derived from a primary human prostatic carcinoma. *Cancer Res* **54**: 6049–6052

Werbajh S, Nojek I, Lanz R and Costas MA (2000) RAC 3 is a NF-κB co-activator. *FEBS Letter* **485**: 195–199

Wilson CM and McPhaul MJ (1994) A and B isoforms of the androgen receptor are present in human genital skin fibroblasts. *Proc Natl Acad Sci USA* **91**: 1234–1238

Xu J, Qiu Y, DeMayo FJ, Tsai SY, Tsai MJ and O'Malley BW (1998) Partial hormone resistance in mice with disruption of the steroid receptor co-activator–1 (SRC–1) gene. *Science* **279**: 1922–1925

Xu J, Liao L, Ning G, Yoshida-Komiya H, Deng Chuxia and O'Malley BW (2000) The steroid receptor co-activator SRC-3 (p/CIP/RAC 3/ AIB 1/ACTR/TRAM–1) is required for normal growth, puberty, female reproductive function and mammary gland development. *Proc Natl Acad Sci USA* **97(12)**: 6379–6384

Yeh S and Chang C (1996) Cloning and characterization of a specific co-activator, ARA70 for the androgen receptor in human prostate cells. *Proc Natl Acad Sci USA* **93**: 5517–5521

Yeh S, Miyamato H, Shima H and Chang C (1998) From estrogen to androgen receptor: A new pathway for sex hormones in prostate. *Proc Natl Acad Sci USA* **95**: 5527–5532

*© 2001 Cancer Research Campaign*
